# Recent Progress in Modulation of WD40-Repeat Domain 5 Protein (WDR5): Inhibitors and Degraders

**DOI:** 10.3390/cancers15153910

**Published:** 2023-08-01

**Authors:** Raju Gurung, Darlami Om, Rabin Pun, Soonsil Hyun, Dongyun Shin

**Affiliations:** 1 College of Pharmacy, Gachon University, 191 Hambakmoe-ro, Yeonsu-gu, Incheon 21936, Republic of Korea; raj.gurng@gmail.com (R.G.); darlami.om@gmail.com (D.O.); robkrt@gmail.com (R.P.); 2 College of Pharmacy, Chungbuk National University, 194-31 Osongsaengmyeong 1-ro, Heungdeok-gu, Cheongju-si 28160, Republic of Korea; 3 Gachon Institute of Pharmaceutical Science, Gachon University, 191 Hambakmoe-ro, Yeonsu-gu, Incheon 21936, Republic of Korea

**Keywords:** WD40-repeat domain protein 5 (WDR5), MYC, oncogene, WDR5–MYC interaction inhibitors, targeted protein degradation

## Abstract

**Simple Summary:**

WD40-repeat (WDR) domain proteins play a crucial role in mediating protein–protein interactions that sustain oncogenesis in human cancers. WDR5 has two protein interaction sites, the “WDR5-binding motif” (WBM) site for MYC interaction and the histone methyltransferases SET1 recognition motif “WDR5-interacting” (WIN) site forming MLL complex. Significant efforts have been dedicated to the discovery of inhibitors that target the WDR5 protein. In this review, we address the recent progress made in targeting WDR5 to inhibit MDR5–MYC and MDR5–MLL1 interactions, including its targeted protein degradation and their potential impact on anticancer drug discovery.

**Abstract:**

WD40-repeat (WDR) domain proteins play a crucial role in mediating protein–protein interactions that sustain oncogenesis in human cancers. One prominent example is the interaction between the transcription factor MYC and its chromatin co-factor, WD40-repeat domain protein 5 (WDR5), which is essential for oncogenic processes. The MYC family of proteins is frequently overexpressed in various cancers and has been validated as a promising target for anticancer therapies. The recruitment of MYC to chromatin is facilitated by WDR5, highlighting the significance of their interaction. Consequently, inhibiting the MYC–WDR5 interaction has been shown to induce the regression of malignant tumors, offering an alternative approach to targeting MYC in the development of anticancer drugs. WDR5 has two protein interaction sites, the “WDR5-binding motif” (WBM) site for MYC interaction and the histone methyltransferases SET1 recognition motif “WDR5-interacting” (WIN) site forming MLL complex. Significant efforts have been dedicated to the discovery of inhibitors that target the WDR5 protein. More recently, the successful application of targeted protein degradation technology has enabled the removal of WDR5. This breakthrough has opened up new avenues for inhibiting the interaction between WDR5 and the binding partners. In this review, we address the recent progress made in targeting WDR5 to inhibit MDR5–MYC and MDR5–MLL1 interactions, including its targeted protein degradation and their potential impact on anticancer drug discovery.

## 1. Introduction

**Figure 1:**
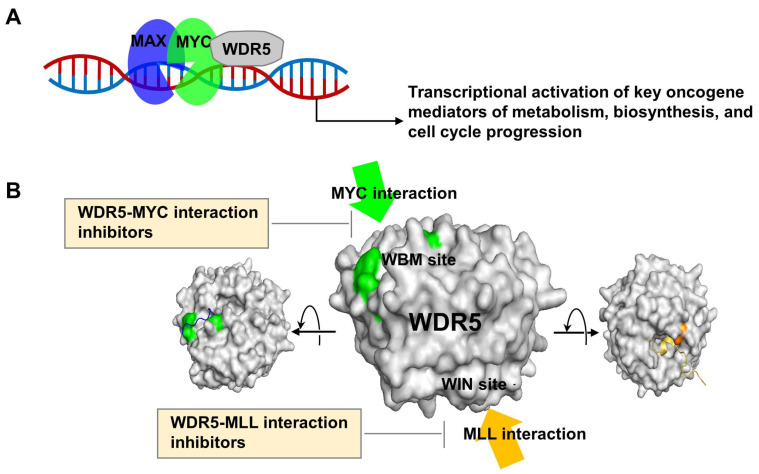
Role of WDR5 in tumorigenesis and its protein interaction partners. (**A**) WDR5 induces transcriptional activation of key oncogenes. (**B**) WDR5 interacts with MYC and MLL proteins on a distinct interface, presenting a promising avenue for the development of therapeutic targets in cancer treatment. Modified X-ray cocrystal structures of WDR5 with MYC peptide (green, PDB: 4Y7R) and WDR5 with Win peptide (orange, PDB: 4ESG).

WD40-repeat-containing protein 5 (WDR5) belongs to the WD repeat protein family, characterized by the presence of repeated WD40 motifs. WD repeats consist of approximately 40 amino acids forming conserved regions that contain the Gly-His and Trp-Asp (GH-WD) repeat motif. These promote the formation of multimeric protein complexes [1]. WDR5, in particular, contains seven WD repeats and interacts with related proteins to be involved in various cellular processes, including gene expression regulation, cell cycle progression, signal transduction, and epigenetic modifications (Figure 1) [1,2,3]. WDR5 has been found to be overexpressed in various cancers, including leukemia [4], breast cancer [5], lung cancer [6], and bladder cancer [7], suggesting its potential as a therapeutic target for cancer treatment [8]. Its interaction with key oncogenes and tumor suppressor proteins affects their function, contributing to cancer development and metastasis. The primary function of WDR5 is its involvement in the assembly and maintenance of histone methyltransferase complexes. Specifically, it is a core component of the MLL (Mixed-Lineage Leukemia) complex (also known as complex of proteins associated with SET1 (COMPASS) or MLL core complex with WDR5, RbBP5, Ash2L, and two copies of DPY-30 (MWRAD_2_)) [9] and MLL1 is a member of the SET1 family of H3K4 methyltransferases [10]. H3K4 methylation depends on the level of assembly of the complex [11]. Thus, MLL1-WRAD2 is a well-known contributor to cancer, especially in poor-prognosis acute leukemias [12]. Additionally, several of these complex members, including WDR5, have recently been implicated in glioblastoma [13]. Recently, WDR5 was also reported to be involved in lymphatic metastasis in bladder cancer through heat shock factor 1 (HSF1)–protein arginine methyltransferase 5 (PRMT5)–WDR5 axis [7]. HSF1 interacts with PRMT5, and finally, the interaction results in an increase in H3K4me3 by the recruitment of WDR5.

WDR5 also interacts with MYC, a well-known oncogene involved in cell growth and proliferation (Figure 1) [14]. This interaction enhances MYC’s transcriptional activity, promoting MYC-driven tumorigenesis. WDR5 interacts with MYC as a major determinant of MYC recruitment to chromatin. Thus, WDR5 induces transcriptional activation of key oncogenes. MYC protein is tightly controlled through multiple checkpoint mechanisms in normal cells, such as apoptosis, cellular senescence, and proliferative arrest [15]. However, the loss of regulatory mechanisms of MYC initiates cancer by modulating the expression of genes related to cell growth and proliferation, as well as inducing changes in the cancer microenvironment [15,16]. Targeted therapy, which aims to control proteins involved in cancer cell survival, is an effective approach in many human cancers. Although the MYC oncoprotein is considered a promising target for anticancer therapy, it is poorly defined pocket has made it challenging to design structure-based inhibitors. Recently, WD-repeat-containing protein 5 (WDR5) has been identified as a specific binding partner of the conserved region of MYC [16]. This discovery has led to alternative strategies for targeting the MYC protein [14,17,18,19,20,21,22]. For example, specific inhibition of the WDR5–MYC interaction has shown a reduction in tumor malignancy, as the function of the MYC transcription factor relies on WDR5 for gene recognition. By targeting WDR5, it is possible to disrupt the activation of MYC and potentially limit cancer progression [23]. 

## 2. WDR5 Inhibitors

### 2.1. WDR5-MYC Interaction Inhibitors

**Figure 2:**
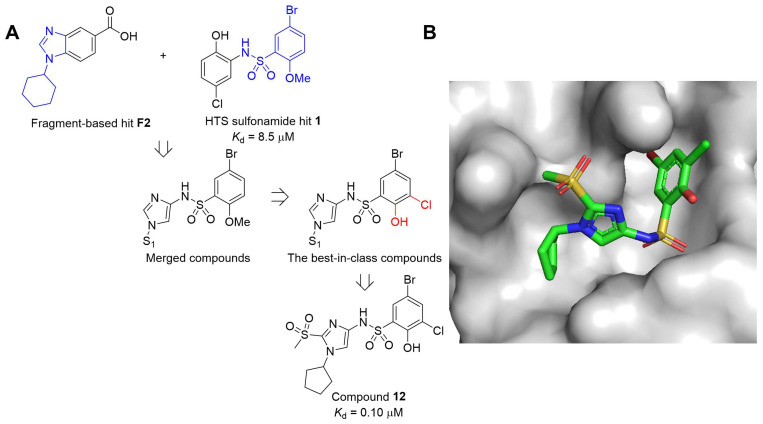
Compound **12** as a WDR5–MYC interaction inhibitor. (**A**) Combining the HTS sulfonamide hit **1** with fragment-based hit **F2** results in merged compounds. Optimization of the merged compounds by incorporation of the phenol and chlorine sulfonyl ring of previously reported best-in-class compounds piece results in improved compounds. Incorporation of methyl sulfone group onto C-2 of the imidazole leads to the strongest binder, compound **12**. (**B**) X-ray cocrystal structure of compound **12** (green, PDB: 6UOZ) bound to WDR5.

Fragment-based screening by the NMR spectroscopy method suggested small molecules, including sulfonamide derivatives, as inhibitors of WDR5-MYC interactions [19,20]. In a recent study by J. D. Macdonald and colleagues, it was reported that small-molecule biaryl sulfonamides disrupt WDR5–MYC interaction [19]. Further modification process was performed to enhance the drug-like properties for in vivo studies by the same group [20]. S. C. Simon and coworkers obtained the NMR spectrum of ^15^N-labeled WDR5 in complex with unlabeled MYC “MbIIIb” peptide. A screening of approximately 14,000 compound fragments from an in-house library was conducted against WDR5 using NMR spectroscopy. Finally, they identified compound **12**, a sulfonamide merged fragment hit compound with a potent binding affinity (*K*_d_ = 0.1 μM, Figure 2). Binding experiments confirmed the robust disruption of the WDR5–MYC interaction in cell lysates. Co-IP studies revealed an approximate fourfold reduction in WDR5–MYC interaction. As a result, compound **12** was demonstrated to decrease the localization of MYC to chromatin at loci where the recruitment of MYC depends on WDR5. Consequently, the authors propose that compound **12** could be further studied for the development of therapeutic agents targeting MYC-driven cancers.

**Figure 3:**
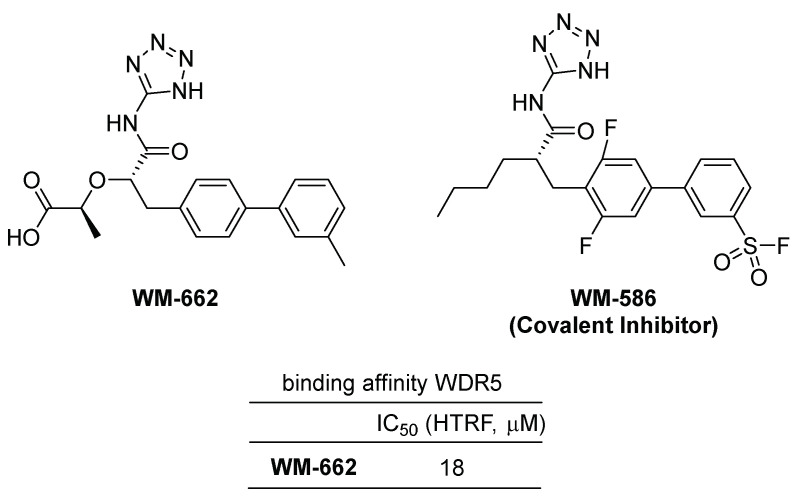
Chemical structure and inhibitory efficiency of **WM-662** and the structure of **WM-586** as WDR5–MYC interaction inhibitors. The IC_50_ value of WM-662 was determined to be 18 μM using HTRF assay. **WM-586**, the first covalent inhibitor, was developed through a structural optimization process of **WM-662**.

In a recent publication, Ding et al. identified **WM-662** as a compound that disrupts the WDR5-MYC interaction by binding the WDR5 binding motif (WBM) of WDR5 (Figure 3) [18]. Using a Novartis in-house library of approximately 330,000 compounds, a high throughput screening was conducted using a competitive homogeneous time-resolved fluorescence (HTRF) assay to identify **WM-662**, which exhibited moderate activity with an IC_50_ value of 18 μM. The crystallographic structural study of the WDR5-**WM-662** complex led to the development of **WM-586**, the first covalent inhibitor of WDR5, through a structure–activity relationship (SAR) study and optimization process (Figure 3). A sulfonyl fluoride-based probe was utilized by introducing a sulfonyl fluoride (SF) group to the phenyl moiety, enabling the generation of covalent adducts with the target protein WDR5 [24]. HTRF measurements of **WM-586**′s IC_50_ values demonstrated time-dependent inhibition, indicating efficient covalent bond formation. The covalent bond formation between **WM-586** and WDR5 was confirmed through mass spectrometry (MS) and liquid chromatography–tandem mass spectrometry (LC-MS/MS) peptide mapping, which revealed that the primary binding residue is mostly Lys250 of WDR5 [18]. 

### 2.2. WDR5-MLL1 Protein–Protein Inhibitors

#### 2.2.1. Peptide and Peptidomimetic Inhibitors of WDR5–MLL1 Interactions

**Figure 4:**
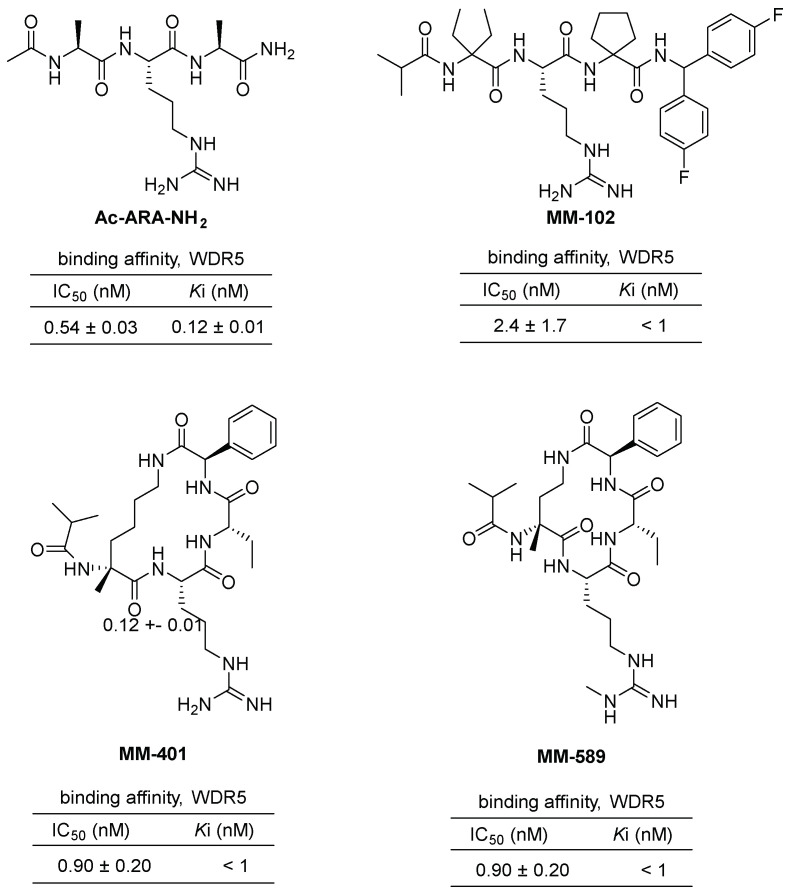
Chemical structures and their inhibitory efficiencies of **Ac-ARA-NH_2_**, **MM-102**, **MM-401**, and **MM-589** as peptide (or peptidomimetic) inhibitors of WDR5–MLL1 interactions. Inhibitory efficiencies, IC_50_ values, against histone H3 lysine 4 (H3K4) methyltransferases (HMT) of reconstituted MLL1 core complex were measured with scintillation counter assay. The fully reconstituted in vitro HMT assay is composed of recombinant MLL1, WDR5, RbBP5, and ASH2L proteins.

The protein–protein interaction between WDR5 and MLL1 plays a critical role in acute leukemia. Consequently, several inhibitors have been developed to mimic this interaction. The first among these is the tripeptide Ac-ARA-NH_2_ (Figure 4), designed by Shaomeng Wang’s group, with a K_i_ value of 120 nM [25]. Through further optimization, the peptidomimetic **MM-102** (Figure 4) was identified, exhibiting an IC_50_ value of 2.4 nM. **MM-102** specifically inhibits cell growth in leukemia cells expressing MLL1 fusion proteins. Analysis of the cocrystal structure of **MM-102** in complex with WDR5 revealed a stronger hydrophobic interaction than its natural ligand, the MLL1 Win peptide. Utilizing this structure, the cyclized peptidomimetic inhibitor **MM-401** (Figure 4) was designed, demonstrating potent inhibition of MLL1 H3K4 methyltransferase (HMT) activity with an IC_50_ value of 0.9 nM and high specificity [26]. It also exhibited specific cytotoxicity against MLL1-dependent leukemia cells. Notably, the inhibition of MLL1 with **MM-401** was shown to play a predominant role in transcriptome regulation in MLL1-dependent leukemia. Subsequently, **MM-589** (Figure 4), a peptidomimetic inhibitor containing monomethylated guanidine, was developed and exhibited cellular potency more than 40 times greater than **MM-401** [27,28,29]. The cocrystal structure **MM-589** with WDR5 further supports its strong inhibition activity. Overall, these inhibitors have demonstrated the potential to mimic the WDR5–MLL1 interaction and inhibit cell growth in leukemia cells. Further research is necessary to evaluate their potential as therapeutic agents.

#### 2.2.2. Benzamide Scaffold Small Molecule Inhibitors of WDR5–MLL1 Interactions

**Figure 5:**
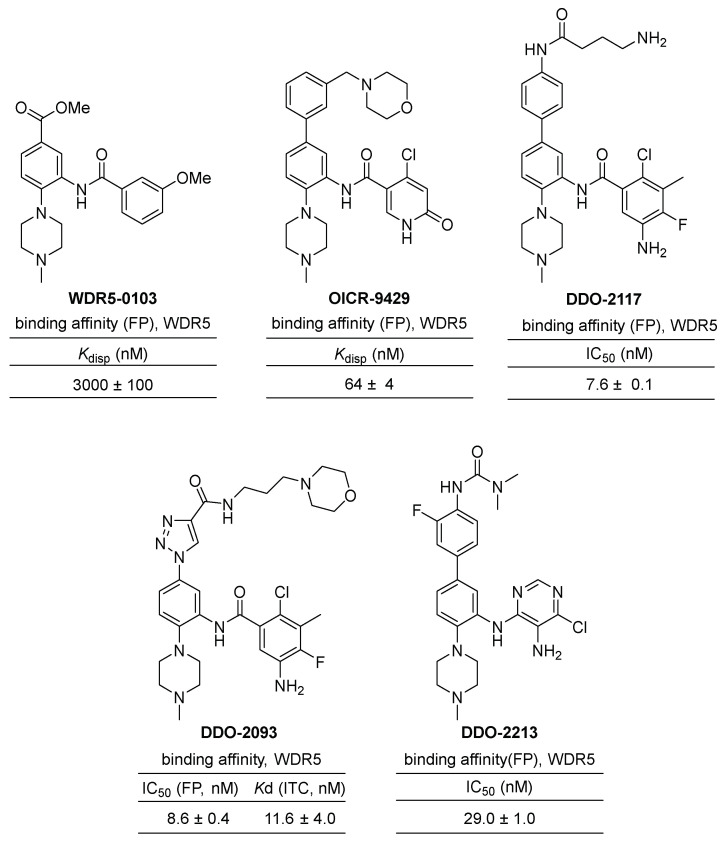
Chemical structures and binding affinities (or inhibitory efficiencies) of **WDR5-0103**, **OICR-09429**, **DDO-2217**, **DDO-2093**, **DDO-2213** as benzamide scaffold small molecule inhibitors of WDR5–MLL1 interactions. Binding affinities were measured by competitive fluorescence polarization assays or ITC experiments.

The discovery of small-molecule inhibitors targeting WDR5–MLL interactions has made significant progress in recent years [30,31,32]. A high-throughput screening of a 16,000-molecule library using fluorescence polarization (FP) identified only one strong competitor of the WIN peptide, **WDR5-0101**, which bears a benzamide scaffold. Further screenings of databases containing 6 million similar chemical structures led to the identification of two additional compounds, **WDR5-0102** and **0103**, both sharing a benzamide scaffold (Figure 5). **WDR5-0102** exhibited a binding affinity of a *K*_d_ = 4.0 μM to WDR5, while **WDR5-0103** showed a higher affinity with *K*_d_ = 0.45 μM. In an inhibition assay of MLL1 HMT activity, **WDR5-0103** specifically and dose-dependently decreases the catalytic activity of both the trimeric and tetrameric complexes of MLL1. Crystal structures of **WDR5-0103** in complex with WDR5 (PDB code: 3SMR and 3UR4) revealed that the inhibitor directly competes with the MLL1 peptide, disrupting the WDR5–MLL1 interaction by occupying the central cavity (P2 pocket) that normally accommodates an arginine side chain of interacting peptides. This structural information explained the improved potency of **WDR5-0103** and provided a basis for the further development of the inhibitors targeting WDR5–MLL1 protein–protein interactions. 

**OICR-9429** is a potent small-molecule inhibitor of the WDR5–MLL1 interaction, discovered through further optimization of an initial phenyl benzamide hit, **WDR5-0102** (Figure 3). **OICR-9429** has been utilized as a high-quality chemical probe in various studies investigating the mechanism of WDR5–MLL1 interaction. For example, **OICR-9429** selectively inhibits proliferation and induces differentiation of human acute myeloid leukemia (AML) cells expressing C/EBPα p30, a mutation present in 9% of AML patients [32]. Furthermore, it selectively inhibits the proliferation of tumor cells expressing gain-of-function (GOF) mutant p53 resulting in increased histone methylation [33]. It has also been shown to suppress metastasis and PDL1-based immune evasion and increase the chemosensitivity of cisplatin in muscle-invasive bladder cancer [34] and advanced prostate cancer [35]. Additionally, it reduces neuroblastoma cell proliferation by blocking the formation of the WDR5–MYCN complex, which is another isomeric family of MYC [36]. The complex crystal structure of WDR5 with **OICR-9429** reveals van der Waals contacts with A47, L321, and I305, as well as a parallel π-stacking interaction of the phenyl ring of the C-5 substituent with the side chains of F133 and Y191 [32,37]. These interactions make **OICR-9429** a powerful inhibitor and an ideal chemical probe for further understanding of WDR5–MLL1 interaction and a potential therapeutic for MLL1-driven acute myeloid leukemias.

The Guo group also investigated the structure–activity relationship based on **W-26** (*K*_d_ = 206 nM), a 4-amino-biphenyl inhibitor [31]. Focused optimization of biphenyl inhibitors led to the development of a potent WDR5-MLL1 inhibitor, **DDO-2117**, with a *K*_d_ value of 13.6 nM and an IC_50_ value of 0.19 μM in HMT screening [38]. 

In 2021, the Guo group reported a linear scaffold hopping approach for the discovery of new phenylalkynyl inhibitors. They synthesized a series of phenyltriazoles through further derivatization using click chemistry and found that compound **DDO-2093** exhibited the strongest binding affinity with a *K*_d_ value of 11.6 nM and an IC_50_ value of 8.6 nM [39]. Remarkably, **DDO-2093** demonstrated selectivity against leukemia cell lines. Additionally, it showed a well-balanced water solubility and cell permeability, and no obvious toxicity was found in normal mice. In vivo assays in the MV4-11 xenograft mouse model further demonstrated that **DDO-2093** significantly reduced tumor volume by inhibiting MLL1 HMT activity and downstream signaling gene expression. 

The same research group discovered **DDO-2213**, an orally active and potent inhibitor of WDR5–MLL1 protein–protein interaction [40]. Through a scaffold-hopping strategy from the initial benzamide inhibitor **WDR5-0103**, they conducted a comprehensive structure–activity relationship study was carried out to identify **DDO-221** as a strong inhibitor. **DDO-221** exhibited an IC_50_ of 29 nM in a competitive fluorescence polarization assay and a *K*_d_ value of 72.9 nM for the WDR5 protein. It selectively inhibited MLL histone methyltransferase activity and the proliferation of cells harboring MLL translocation. Furthermore, **DDO-2213** demonstrated optimal metabolic stability in mouse and human microsomes, along with sufficient pharmacokinetic properties for oral administration. The inhibitor effectively suppressed the growth of MV4-11 xenograft tumors in mice following oral administration. Importantly, this study represents the first verification of the in vivo efficacy of a small molecule targeting the WDR5–MLL1 protein–protein interaction.

#### 2.2.3. Acridine-1,8-Dione Scaffold Small Molecule Inhibitors of WDR5–MLL1 Interactions

Ye et al. recently reported the discovery of novel acridine-1,8-diones as inhibitors of WDR5–MLL1 interaction [41]. They employed an FP-based high throughput screening to discover small-molecule inhibitors that effectively target the WDR5–MLL1 interaction. Subsequently, nuclear magnetic resonance (NMR) assays were conducted to validate the direct binding between the identified compound and WDR5 and resulting in the development of **DC-M5-2** as an inhibitor of WDR5–MLL1 interaction. 

**Figure 6:**
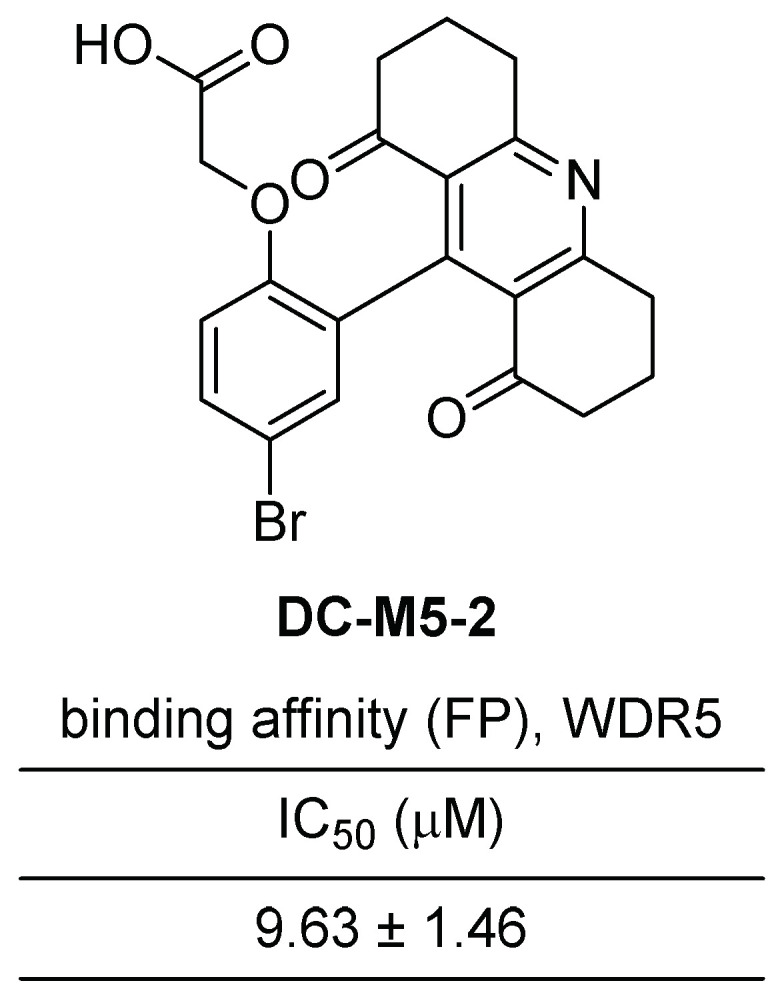
Chemical structure and binding affinity of **DC-M5-2** as acridine-1,8-dione scaffold of WDR5–MLL1 interaction.

**DC-M5-2** is characterized as a 9-phenoxy-decahydroacridine-1,8-dione compound, featuring an acid functionality at the ortho position of the phenyl group (Figure 6). The inhibitory activity of **DC-M5-2** was demonstrated with an IC_50_ value of 9.63 μM. To gain further insights into the binding modes and interaction mechanisms between **DC-M5-2** and WDR5, a molecular docking study was performed. The results revealed that **DC-M5-2** establishes several hydrophobic contacts with surrounding residues, including S49, D107, Y131, Y260, F133, I305, and L321. Additionally, the carboxyl group of **DC-M5-2** participates in the formation of a hydrogen bond network with Y191 and C261. 

#### 2.2.4. Benzyl Benzamides Scaffold Small Molecule Inhibitors of WDR5–MLL1 Interactions 

**Figure 7:**
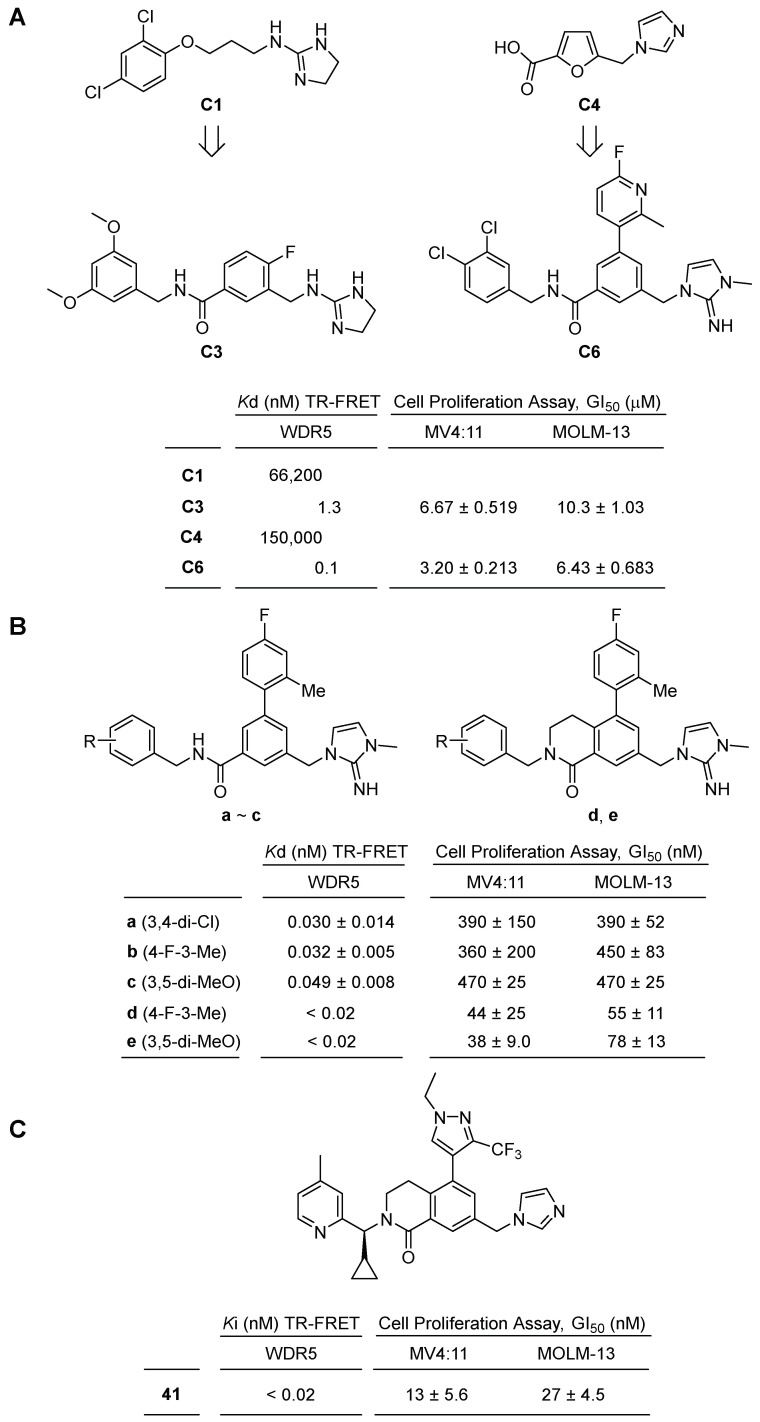
Benzyl benzamides scaffold as WDR5–MLL1 interaction inhibitors. (**A**) Optimization of inhibitor using a fragment library screening using the HMQC NMR method. (**B**) Further modifications of **C6**. (**C**) Inhibitory binding affinity Ki value using TR-FRET competition assay and half-maximal growth inhibition (GI_50_) value of compound **41**.

In 2019, novel benzyl benzamides were reported as inhibitors of the WIN site of WDR5 with picomolar affinity [42]. Aho, E. R. et al. conducted a fragment library screening using the HMQC NMR method, screening approximately 13,800 compounds to identify small molecule hits that bind the WIN site. Among the fragment hits, compound **C1**, a 2- alkylaminoimidazoline, exhibited a *K*_d_ value of 66 μM. Further optimization led to the development of benzyl benzamide **C3**, which displayed improved binding affinity with a *K*_d_ value of 1.3 nM (Figure 7A). Additionally, another fragment hit, **C4**, initially showed a *K*_d_ value of 150,000 nM but underwent structural evolution to become the second-generation WIN site inhibitor **C6**, exhibiting picomolar binding affinity (*K*_d_~100 pM), representing a remarkable 1,500,000-fold improvement in binding affinity. 

In vitro evaluations of **C3** and **C6** focused on their ability to inhibit cell growth against two representative leukemia cell lines, MV4:11 and MOLM-13. Compound **C3** demonstrated moderate growth inhibition, with IC_50_ values of 6.67 μM against MV4:11 and 10.3 μM against MOLM-13. Compound **C6** also exhibited moderate growth inhibition, with IC_50_ values of 3.20 μM against MV4:11 and 6.43 μM against MOLM-13 (Figure 7A).

In a subsequent article in 2020, a structure-based optimization study was conducted on inhibitor **C6** to discover more potent and selective WDR5 inhibitors [43]. Tian, J. et al. made modifications to the compound, starting by replacing the upper 4-fluoro-2-methylphenyl with 4-fluoro-4-methylphenyl. They then introduced diverse substituents to the benzyl group and found that 3,4-chlorobenzyl, 4-fluoro-3-methylbenzyl, and 3,5-dimethoxy derivatives displayed potent binding affinities with 0.030, 0.032, 0.049 nM, respectively (Figure 7B).

To further enhance potency and improve drug-like properties, the authors employed a ring-closing strategy, converting the benzamide into dihydroisoquinoline. This modification significantly increased both binding affinity and cell growth inhibitory activity. The dihydroisoquinoline derivatives compounds **d** and **e** exhibited binding affinities of less than 0.02 nM and half-maximal growth inhibition (GI_50_) values of 44 and 38 nM against MV4:11 cell lines, demonstrating potency approximately ten times greater than that of the benzamide derivatives (Figure 7B).

However, despite its robust binding and inhibitory activity of compound **e** towards the WDR5 WIN-site, the most promising candidate exhibited certain limitations, including low solubility, poor permeability, and insufficient pharmacokinetic profiles [44]. In order to gain further understanding of the binding mechanisms of WDR5 and compound **e**, Teuscher, K. B. et al. obtained the crystal structure of the WDR5-compound **e** complex. This structural analysis revealed that the 4-fluoro-2-methylphenyl group binds to hydrophobic subsites and exhibits potential tolerance toward heterocycles. Therefore, they substituted the 4-fluoro-2-methylphenyl group with various six- and five-membered heterocycles, such as substituted pyridines, isoxazoles, and pyrazoles. The derivatives displayed binding affinities comparable to compound **e**. Notably, 2-methylpyridin-3-yl, 2,5-dimethylpyridin-4-yl, 1-methyl-3-(trifluoromethyl)pyrazol-4-yl, and 1-ethyl-3-(trifluoromethyl)pyrazol-4-yl exhibited good binding affinities of less than 0.02 nM. 

Furthermore, the optimization of the 2-iminoimidazole moiety involved the introduction of other nitrogen-containing five-membered heterocycles, including substituted imidazoles, pyrazoles, and triazoles. Among the derivatives, 10midazole-1-yl and 2-methylimidazol-1-yl analogs showed binding affinities of less than 0.02 nM. Finally, the benzyl group was further optimized by transforming it into a substituted pyridine with a benzylic alkyl group, resulting in compound **41** (Figure 7C). This compound exhibited a cLogP of 3.6, a kinetic solubility of 66 μM, a binding affinity of less than 0.02 nM, and a GI_50_ value of 17 nM against MV4:11. 

## 3. WDR5 Degraders

**Figure 8:**
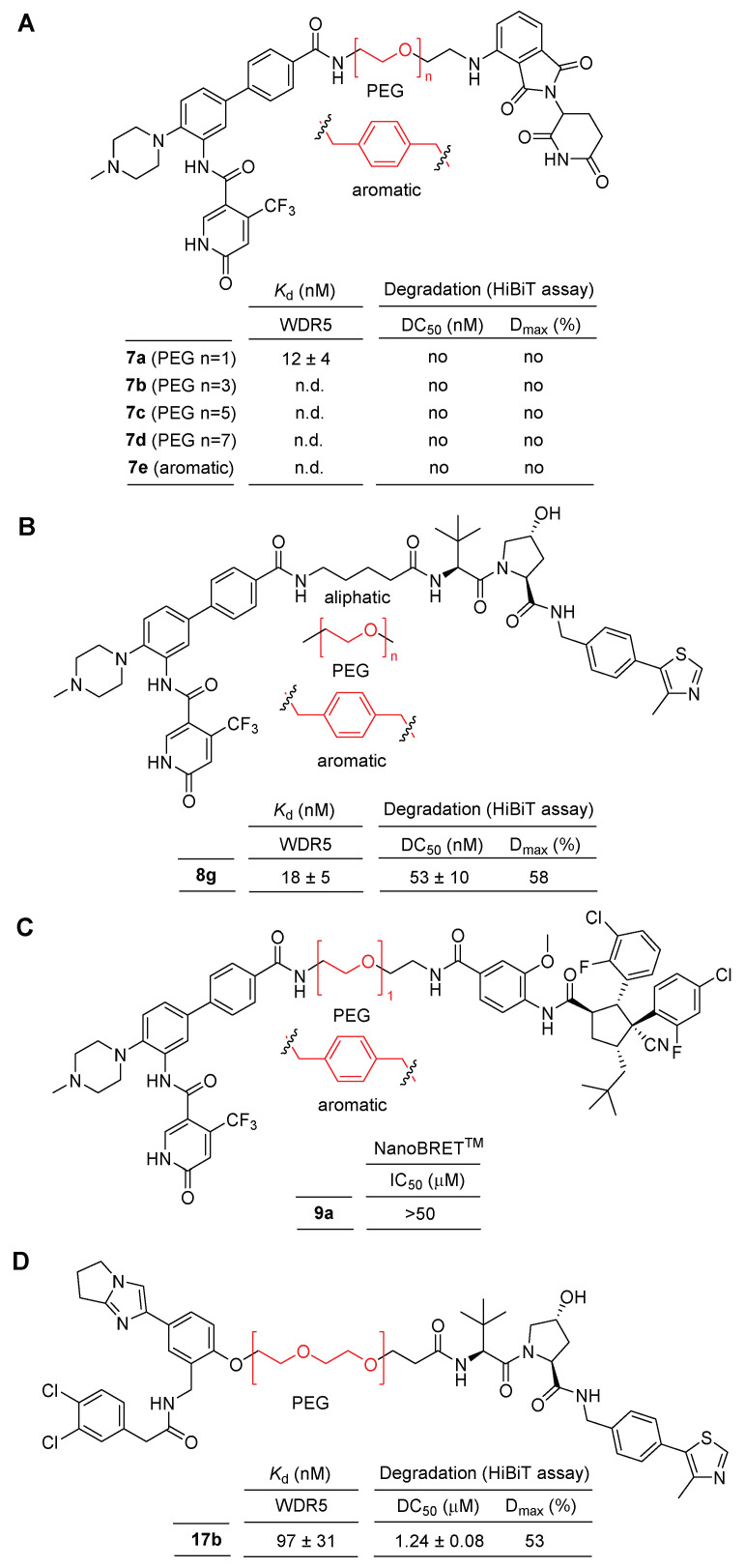
Dual WDR5 and Ikaros degraders. (**A**) A combination of **OICR-9429** and pomalidomide was synthesized, incorporating PEG and aromatic linkers. (**B**) A combination of **OICR-9429** with a VHL ligand, utilizing aliphatic, PEG, and aromatic linkers. (**C**) A combination of **OICR-9429** with an MDM ligand, incorporating PEG and aromatic linkers. (**D**) a combination of a pyrroloimidolylbenzene scaffold and a VHL ligand attached by a PEG linker.

Targeted protein degradation represents a burgeoning class of therapeutic interventions, with the PROTAC (PROteolysis Targeting Chimeras) technology occupying a prominent position in drug discovery [45]. PROTAC is a dual-function molecule consisting of a small molecule binder for a protein of interest (POI) and a ligand for the E3 ubiquitin ligase. Upon binding to POI, PROTAC can recruit E3 ligase for POI ubiquitination, which is proteolytically degraded by the ubiquitin-proteasome system (UPS). Recently, numerous biotechnology companies are focusing their efforts on the exploration and development of PROTAC-based drugs. Thus, the pivotal role of WDR5 as a scaffolding protein has attracted considerable interest in pursuing targeted degradation strategies targeting WDR5. As some reports have illuminated pharmacologic and expression-level suppression of WDR5, it was explored whether selective functional protein degradation is more efficient compared to the generation of non-functional proteins [32,46]. In 2021, Dölle et al. reported the pioneering design and synthesis of WDR5 degraders based on PROTACs [47]. These PROTACs were designed by incorporating known WDR5 inhibitors, namely **OICR-9429** and pyrroloimidazole scaffold inhibitors [32,37,48], as binders for the protein of interest (POI), and employing representative E3 ligases, CRBN, VHL, and MDM229, as the degradation platforms. In the initial series of WDR5-targeting PROTACs, a combination of **OICR-9429** as a WDR5 binder and pomalidomide as a ligand for CRBN was synthesized, incorporating PEG and aromatic linkers (Figure 8A). However, in the degradation assay, none of the PROTAC compounds resulted in the elimination of WDR5, while compound **7a** showed 12 nM of *K*_d_ value.

Subsequently, a new series of PROTAC compounds were designed and synthesized by combining **OICR-9429** as a WDR5 binder with a VHL ligand, utilizing aliphatic, PEG, and aromatic linkers (Figure 8B). A total of nine PROTAC compounds were prepared. The aliphatic and aromatic linked PROTACs exhibited moderate to good binding affinity, with compound **8g**, featuring a four-carbon linker, displaying the highest degradation activity (DC_50_ = 53 nM). However, the maximum degradation efficacy achieved was relatively low, with 58% of D_max_ (Figure 8B). Additionally, three PROTAC compounds were designed and synthesized by combining **OICR-9429** as a WDR5 binder with an MDM ligand, incorporating PEG and aromatic linkers (Figure 8C). Unfortunately, these compounds did not exhibit significant binding affinity towards WDR5, determined by the NanoBRET assay.

A series of WDR5-PROTAC was designed and synthesized, incorporating a pyrroloimidolylbenzene scaffold as the WDR5 binder and a VHL ligand attached by PEG linkers (Figure 8D). Among the seven PROTAC compounds tested, only one PROTAC with two PEG linkers, compound **17b**, exhibited moderate binding affinity and mild degradation activity (DC_50_ = 1.24 nM). 

Although various WDR5-PROTAC degraders were developed by employing different WDR5 binders and E3 ligase ligands, it was observed that the degradation activity is highly influenced by the type and length of the linker, as well as the choice of E3 ligase ligand. PROTACs based on the VHL E3 ligase platform demonstrated relatively better degradation activity. However, limited structural variations were explored regarding the exit vectors of WDR5 binders, E3 ligase ligands, and linkers.

**Figure 9:**
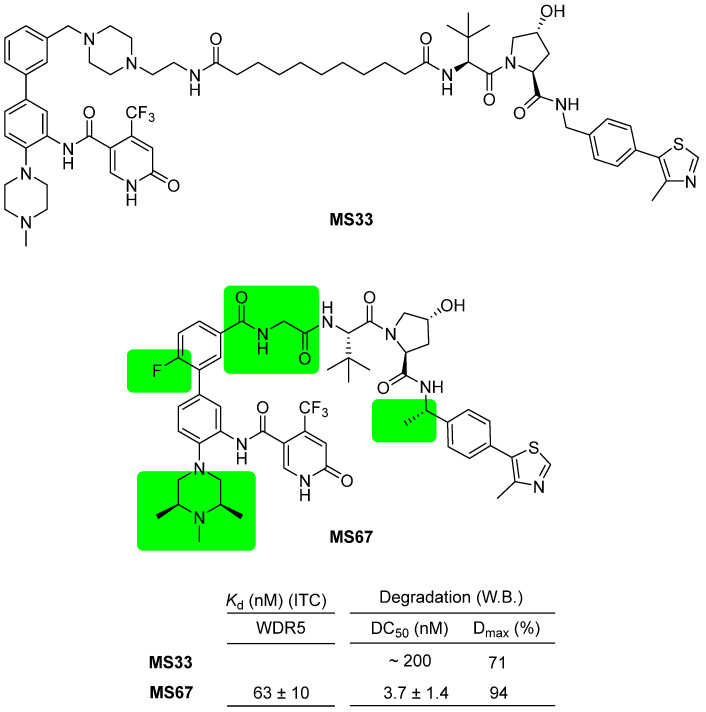
WDR5 PROTAC degraders, **MS33** and **MS67**, composed of phenylbenzamide-scaffolded WDR5 binders and VHL ligands. The difference between **MS33** and **MS67** is highlighted in green.

Selective PROTAC degraders targeting WDR5 have been reported and demonstrated to effectively suppress tumor growth in vivo and improve survival rates in an AML-PDX xenograft model. Two PROTAC degraders, namely **MS33** and **MS67**, composed of phenylbenzamide-scaffolded WDR5 binders and VHL ligands, were introduced (Figure 9) [39]. These degraders exhibit structural variations compared to previous WDR5 PROTACs, specifically in the exit vector of the phenylbenzamide-scaffolded WDR5 binder, which is positioned meta to the phenyl group. **MS33** and **MS67** display distinct structural differences as well. The linking chemistry between the two PROTACs differs; **MS33** employs an alkyl linker, while **MS67** utilizes an amide linker. Additionally, the WDR5 binder was optimized from *N*-methylpiperazine in **MS33** to 2,6-dimethyl-*N*-methylpiperazine in **MS67**. Importantly, the two PROTACs are distinguished by their linkers; **MS33** possesses a relatively long piperazinylalkyl linker, while **MS67** has a very short or no linker. X-ray crystallographic data of the two PROTACs revealed that the structures of the ternary complexes differ completely based on the linker length. Ultimately, the short-linked **MS67** exhibited superior binding affinity and higher degradation efficacy. **MS67** demonstrated degradation values of 3.7 nM of DC_50_ value and 94% for D_max_ against wild-type WDR5, while it exhibited no degradation activity against WDR5 D172A mutant and reduced activity against WDR5 Y191A mutant. **MS67** exhibited potent growth suppression of AML cells from deidentified patients in vitro and displayed efficacy in an MLL-AF9^+^ AML PDX-xenograft model, leading to prolonged survival rates without any significant loss of body weight.

**Figure 10:**
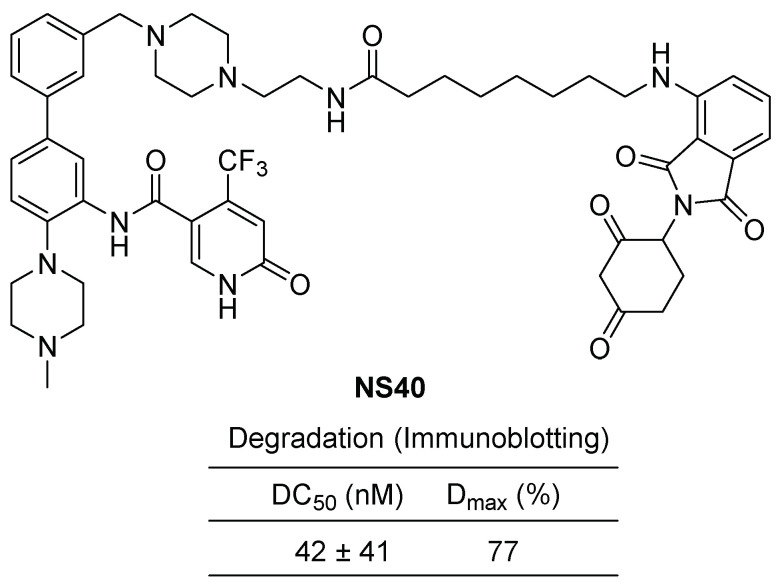
Dual WDR5 and Ikaros 1/3 Degrader, **NS40**, linking phenylbenzamide as a WDR5 binder and lenalidomide as a CRBN ligand using a piperazine-containing alkylamide linker.

In 2022, the same research group published a study on a dual degrader targeting both WDR5 and CRBN neo-substrates [49]. Thalidomide and its derivatives, such as lenalidomide and pomalidomide, have been demonstrated as effective degraders against ikaros and aiolos, leading to their utilization as anticancer drugs for multiple myeloma. The dual degrader **NS40** was designed by linking phenylbenzamide as a WDR5 binder and lenalidomide as a CRBN ligand using a piperazine-containing alkylamide linker (Figure 10). **NS40** was found to induce the degradation of WDR5 through the PROTAC mechanism and ikaros1/3 through the molecular glue mechanism. **NS40** exhibited successful inhibition of tumor cell growth and demonstrated efficacy in a PDX mouse xenograft model.

## 4. Conclusions

Until now, peptidomimetics and small molecules targeting the WDR5 protein–protein interaction (PPI) have been developed as anticancer strategies. Those WDR5 inhibitors reduce the proliferation of cancer cells. However, their evaluation has primarily been limited to cellular studies due to their non-optimized drug-like properties. Some in vivo animal studies of WDR5-MLL1 inhibitors have been performed, and no clinical studies have been reported so far. The efficacies and affinities of these inhibitors still require validation in animal models and subsequent clinical trials. Furthermore, the inhibition of the WDR5 PPI may potentially lead to cytotoxicity and side effects due to the nonselective interference with normal cellular functions. It has also been reported that excessive targeted protein degradation can lead to on-target-mediated side effects similar to gene knockdown approaches. Thus, it is imperative to assess the safety profiles of these inhibitors in future investigations. However, it is still noteworthy that a few WDR5 targeting compounds were reported as efficient with no toxicity. For example, OICR-9429, a small molecule antagonist, has low toxicity to normal cells and selectively inhibits only AML cells with upregulated *C/EBPα*-mutant p30 isoform. Some WDR5 inhibitors have shown dose-dependent in vivo antitumor efficacy without toxicity by oral dosing. WDR5 PROTACs have recently shown in vivo efficacy in a mouse MV4:11 xenograft model. 

Finally, the development of WDR5 inhibitors and degraders is at an early stage but still with great potential to target several cancers. 

## Data Availability

Not applicable.
